# Exposing a novel genetic interaction between *unc-33/CRMP* and *hmp-2/β-catenin* during *Caenorhabditis elegans* embryogenesis

**DOI:** 10.17912/micropub.biology.000286

**Published:** 2020-07-30

**Authors:** John Garzanelli, Stephanie Maiden

**Affiliations:** 1 Biology Department, Truman State University, Kirksville, MO, 63501

**Figure 1. Percentage of embryos that failed to hatch after feeding RNAi knockdown in wildtype (A) or unc-33(e204) homozygous animals (B):**
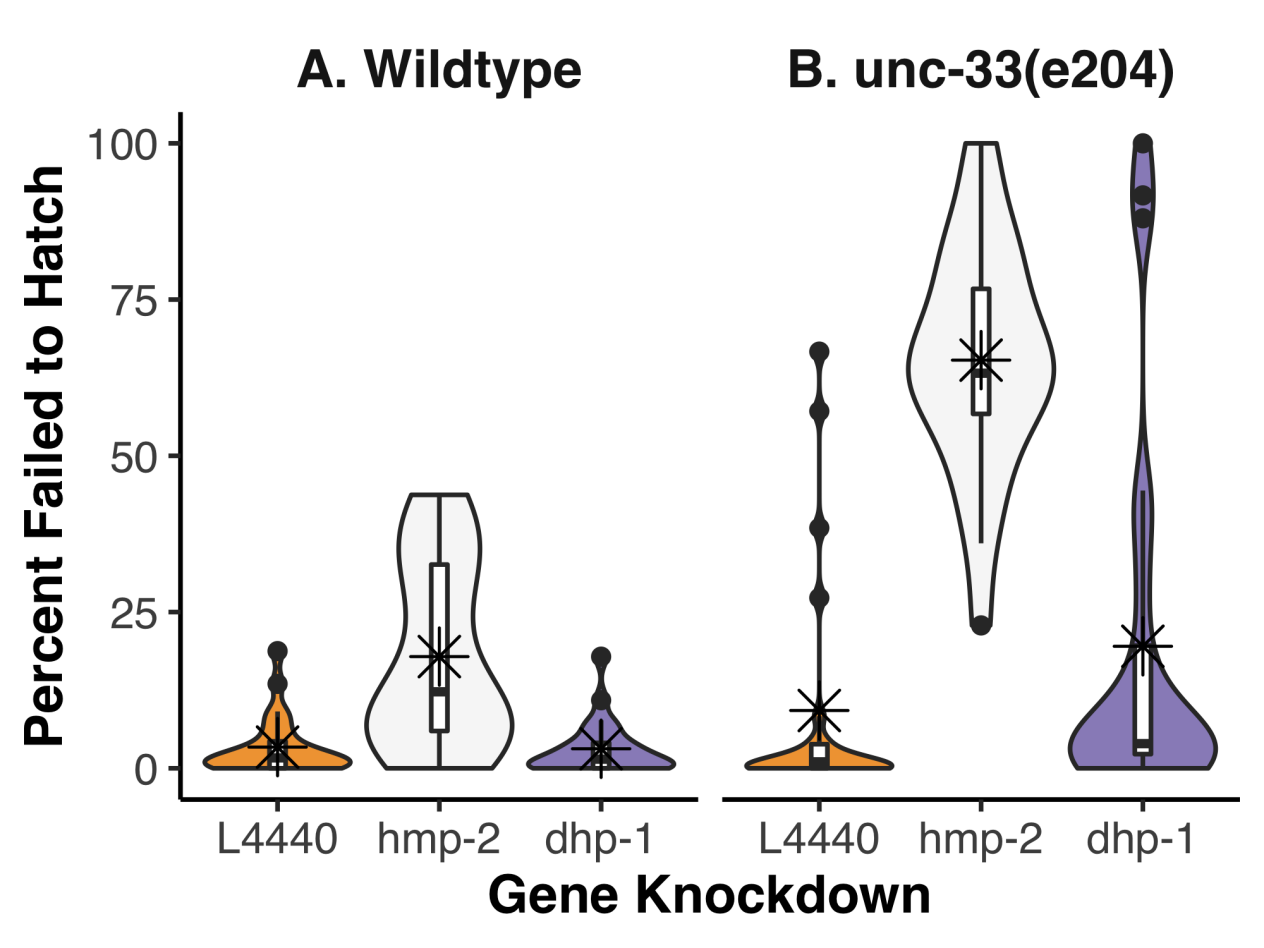
Box limits indicate the 25th and 75th percentiles as determined by R software; whiskers extend 1.5 times the interquartile range from the 25th and 75th percentiles, outliers are represented by dots. Horizontal bars indicate the medians, asterisks indicate the means, and polygons represent density estimates of data and extend to extreme values. From left to right in the plot, n=24, 23, 24, 23, 23, 24, where each *n* is the percentage that failed to hatch from a single hermaphrodite. Standard deviations from left to right are 4.8, 14.8, 4.3, 19.2, 18.4, 30.9.

## Description

Microtubules are critical to a number of vital cellular processes, including cell division, intracellular transport, cell movement, and cell structure. Microtubule-associated proteins (MAPs) facilitate these various microtubule functions by regulating dynamic instability, crosslinking, and trafficking, among others (Alfaro-Aco and Petry 2015). There are over 60 conserved MAPs in *Caenorhabditis elegans*, many with essential functions (Oegema 2006; Lacroix *et al.* 2014; Rose and Gonczy 2014; Quintin *et al.* 2016). Not surprisingly, microtubules and MAPs are required during early *Caenorhabditis elegans* embryogenesis when cell divisions predominate (Oegema 2006; Rose and Gonczy 2014). Temporally-controlled microtubule disruption by either drugs or transgenic constructs can bypass these early requirements and have shown that microtubules are also necessary for proper epidermal morphogenesis during mid-embryonic development (Williams-Masson *et al.* 1998; Quintin *et al.* 2016). However, it is still unclear what complement of MAPs function during this stage of development to pattern the overall microtubule network and control dynamic instability.

*C. elegans* UNC-33 is an ortholog to collapsin response mediator proteins (CRMPs), a family thought to regulate the dynamic instability of microtubules (Li *et al.* 1992; Fukata *et al.* 2002; Tsuboi *et al.* 2005; Lin *et al.* 2011). Mutants of *unc-33* exhibit defects in axonal outgrowth and guidance, resulting in paralysis and locomotor defects (Hedgecock *et al.* 1985; Li *et al.* 1992). The dendrites of sensory neurons in these animals have far more microtubules than in wildtype and they are often larger in diameter with other structural defects (Hedgecock *et al.* 1985). In addition to these neuronal effects, homozygous mutant animals of the classic allele, *unc-33(e204)*, also appear shorter and stouter than wildtype animals. While some Dumpy animals are caused by mutations in genes important for cuticle development (Kusch and Edgar 1986), others are caused by mutations in genes important for embryonic epidermal morphogenesis, such as *sma-1* (McKeown *et al.* 1998; Praitis *et al.* 2005), *let-502* (Piekny *et al.* 2000; Gally *et al.* 2009; Quintin *et al.* 2016), and *lin-26* (Ferguson and Horvitz 1985).Two other *C. elegans* gene products, DHP-1 and DHP-2, are almost 70% identical in sequence with UNC-33 and are also evolutionarily related to vertebrate CRMPs (Takemoto *et al.* 2000). *In situ* hybridization showed *dhp-1* mRNA in hypodermal cells from late gastrula to at least 2-fold body elongation, and a GFP-tagged version of DHP-1 was also expressed in the larval hypodermis (Takemoto *et al.* 2000). There are currently no associated phenotypes to *dhp-1*,so we hypothesized that UNC-33 and DHP-1 may be functionally redundant in the epidermis during *C. elegans* embryogenesis.

To determine if any functional redundancy exists between UNC-33 and DHP-1, we used *unc-33(e204)* homozygous animals as a sensitized background and disrupted *dhp-1* expression by feeding RNA interference (RNAi). Because we were specifically interested in potential embryonic effects, we scored the number of embryos that failed to hatch and took this as a percentage of the total progeny (Figure 1). When analyzed by two-way ANOVA, a significant interaction was found between the strains and gene knockdowns (p=2.761e-07). We used post-hoc Tukey’s honest significant test to make all pairwise comparisons; this revealed which of the gene knockdowns interacted with *unc-33(e204)* mutants. As expected for a negative control, the empty feeding vector (L4440) fed to either wildtype or *unc-33(e204)* homozygous animals showed a low level of lethality, 3.4% and 9.3% on average, respectively. These data were not significantly different from each other in a pairwise comparison (p=0.872). In wildtype animals, the positive control, *hmp-2(RNAi),* resulted in 17.9% of embryos that failed to hatch but this was not found to be significantly different to the L4440 control (p=0.068). It is important to note, however, that less than 1% of hatched *hmp-2(RNAi)* animals were normal larva; the vast majority were Hmp with severe body morphology defects. Surprisingly, *unc-33(e204);hmp-2(RNAi)* resulted in 65.3% of embryos that failed to hatch, a significant increase compared to both the negative control in the same strain and *hmp-2(RNAi)* in wildtype (p < 0.001 for both). In both wildtype and *unc-33(e204)* homozygous animals, knockdown of *dhp-1* did not significantly alter the percent of embryos that failed to hatch (3.1% and 19.5%, respectively).

Our hypothesis regarding UNC-33 and DHP-1 was unfortunately not supported by this experiment. While *unc-33(e204)* is considered the reference allele, it is also a weak loss-of-function (Tsuboi *et al.* 2005) and may still provide enough UNC-33 activity to obscure any redundancy with DHP-1 that might be revealed by *dhp-1* knockdown. The unexpected interaction between *hmp-*2 and *unc-33* is intriguing, however, given that our hypothesis focused on the role of UNC-33 and DHP-1 in the embryonic epidermis. Knockdown of *hmp-2/β-catenin* by feeding RNAi was chosen as a positive control because of the reproducible embryonic and larval phenotypes in wildtype. The embryonic lethality and body morphology defects from *hmp-2(RNAi)* are due to the essential role of HMP-2/β-catenin at adherens junctions during epidermal morphogenesis (Costa *et al.* 1998). HMP-2/β-catenin binds the intracellular tail of the transmembrane HMR-1/cadherin, strengthening cell-cell adhesion by simultaneously binding to the actin-binding protein HMP-1/α-catenin (Costa *et al.* 1998; Maiden and Hardin 2011; Maiden *et al.* 2013). During epidermal morphogenesis, a subset of epidermal cells migrates from the dorsal surface towards the ventral midline of the embryo. Once ventral enclosure is completed, actomyosin contractions in the epidermis shorten the cells along the dorsoventral axis while lengthening occurs along the anteroposterior axis, elongating the body of the animal 4-fold by the time of hatching (Chisholm and Hardin 2005). F-actin fails to properly localize to adherens junctions when there is a loss of HMP-2/ β-catenin or HMP-1/α-catenin, producing shorter animals with dorsal humps when the process of body elongation fails (Costa *et al.* 1998; Maiden *et al.* 2013). The phenotypes we observed in wildtype after *hmp-2(RNAi)* by feeding are relatively weak as RNAi by injection, or animals that are homozygous null, results in almost complete embryonic lethality (Costa *et al.* 1998; Sönnichsen *et al.* 2005). Several studies have implicated microtubules in the regulation of cadherin-based adhesions (Aono *et al.* 1999; Stehbens 2006; Meng *et al.* 2008; Maiden *et al.* 2016; Ning *et al.* 2016; Quintin *et al.* 2016), including one in *C. elegans* that showed disrupting microtubules at the 1.5/1.7-fold stage of elongation also perturbed junctional E-cadherin appearance and turnover (Quintin *et al.* 2016). The greater percentage of embryos that failed to hatch after *hmp-*2 knockdown in *unc-33(e204)* compared to wildtype might further support a role for microtubules at adherens junctions and indicate a novel role for UNC-33 in regulating microtubule dynamics during epidermal morphogenesis. Future experimentation using advanced microscopy and additional genetic analyses will be needed to determine how and when this particular genetic interaction impacts embryogenesis.

## Methods

*C. elegans* strains were cultured using standard protocols (Brenner 1974). Strains used include N2 [wildtype Bristol] and CB204 [*unc-33(e204)* IV]. Bacterial clones for feeding RNAi were obtained from the Ahringer library (Kamath and Ahringer 2003). Each gene insert was verified by Sanger sequencing through Eurofins Genomics using the following vector primers: GTCAGTGAGCGAGGAAGCAAC and CTCTTCGCTATTACGCCAGCTG. For knockdown by feeding RNAi, bacterial cultures were plated on NGM plates supplemented with 25 μg/mL carbenicillin, 10 μg/mL tetracycline, and 1 mM IPTG, and incubated for 3 days at room temperature to induce double-stranded RNA. L4 worms at 20 °C were fed bacteria for 48 hours and then singled to individual plates. After laying eggs for approximately 18 hours, the adults were removed and all progeny were counted via a dissecting microscope. After an additional 24 hours, the remaining number of eggs were counted. The violin plot, basic summary statistics, two-way ANOVA Type III, and the post-hoc Tukey analysis were all completed using R.
